# Cerebrospinal Fluid Biomarkers in iNPH: A Narrative Review

**DOI:** 10.3390/diagnostics12122976

**Published:** 2022-11-28

**Authors:** Efstratios-Stylianos Pyrgelis, Fotini Boufidou, Vasilios C. Constantinides, Myrto Papaioannou, Sokratis G. Papageorgiou, Leonidas Stefanis, George P. Paraskevas, Elisabeth Kapaki

**Affiliations:** 1 1st Department of Neurology, School of Medicine, National and Kapodistrian University of Athens, Eginition Hospital, Vass. Sophias Ave. 74, 11528 Athens, Greece; 2 Neurochemistry and Biological Markers Unit, 1st Department of Neurology, School of Medicine, National and Kapodistrian University of Athens, Eginition Hospital, Vass. Sophias Ave. 74, 11528 Athens, Greece; 3 2nd Department of Neurology, School of Medicine, National and Kapodistrian University of Athens, “Attikon” University General Hospital, Rimini 1, 12462 Athens, Greece

**Keywords:** normal pressure hydrocephalus, biomarkers, cerebrospinal fluid, Alzheimer’s disease, vascular dementia, tau proteins, beta amyloid

## Abstract

Idiopathic normal pressure hydrocephalus (iNPH) is a neurological syndrome characterized by the clinical triad of gait disorder, cognitive impairment and urinary incontinence. It has attracted interest because of the possible reversibility of symptoms, especially with timely treatment. The main pathophysiological theory is based on a vicious circle of disruption in circulation of cerebrospinal fluid (CSF) that leads to the deceleration of its absorption. Data regarding CSF biomarkers in iNPH are contradictory and no definite CSF biomarker profile has been recognized as in Alzheimer’s disease (AD), which often co-exists with iNPH. In this narrative review, we investigated the literature regarding CSF biomarkers in iNPH, both the established biomarkers total tau protein (t-tau), phosphorylated tau protein (p-tau) and amyloid peptide with 42 amino acids (Aβ42), and other molecules, which are being investigated as emerging biomarkers. The majority of studies demonstrate differences in CSF concentrations of Aβ42 and tau-proteins (t-tau and p-tau) among iNPH patients, healthy individuals and patients with AD and vascular dementia. iNPH patients present with lower CSF Aβ42 and p-tau concentrations than healthy individuals and lower t-tau and p-tau concentrations than AD patients. This could prove helpful for improving diagnosis, differential diagnosis and possibly prognosis of iNPH patients.

## 1. Introduction

Hydrocephalus is a condition in which an excess of cerebrospinal fluid (CSF)—the organic liquid that surrounds the brain and spinal cord—accumulates within the subarachnoid space and the ventricles of the brain [1,2]. The term hydrocephalus is derived from the Greek words “hydro” meaning water and “cephalus” meaning the head, understood as “water in the brain” in free translation. Hydrocephalus may be the result of increased production, decreased absorption or obstruction of normal CSF flow. There are two main types of hydrocephalus, obstructive or non-communicating and communicating, referring to intraventricular or extraventricular obstruction, respectively [1,2,3].

Normal pressure hydrocephalus (NPH) is a special type of communicating hydrocephalus occurring more frequently in elderly patients. It is classified into idiopathic normal pressure hydrocephalus (iNPH) and secondary, which can be a complication of previous subarachnoid hemorrhage, traumatic brain injury, infection and/or tumor [4,5].

Clinically, iNPH was first described by Hakim and Adams in 1965 as a syndrome characterized by a triad of symptoms, namely gait disorder, cognitive impairment and urinary incontinence [6,7]. Radiologically, it is characterized by enlarged lateral and third ventricles, disproportionally to cortical sulci enlargement, as depicted by brain imaging, either with computed tomography (CT) or magnetic resonance imaging (MRI) [8]. Increased Evans index, temporal horns dilation, periventricular white matter lesions, narrow callosal angle and sulci in the convexity, focally enlarged sulci and dilated Sylvian fissures are additional imaging findings that have been described [9,10,11,12,13,14].

More than 50 years after its initial description, the exact etiopathogenesis of iNPH remains controversial. Various theories attribute the pathogenesis of iNPH to reduced CSF resorption. Some of them suggest that increased venous resistance is the initial “hit”, while others correlate ischemic white matter lesions to deceleration of CSF flow through the extracellular spaces, resulting in a “back-pressure” effect, leading to ventricular enlargement [15]. The common denominator of these studies seems to be the fact that iNPH is caused by a vicious circle of CSF circulation disruption that leads to a deceleration of its absorption and enlargement of the ventricles [5,16].

In recent years, there has been a surge of interest in iNPH, as in many cases this syndrome may be a reversible condition following a ventriculoperitoneal (VP) shunt or third ventriculostomy for those patients predicted to respond. Under this perspective, a great deal of effort has been placed towards early diagnosis and timely treatment. One of the most well-established diagnostic tests and at the same time a prognostic factor of successful response to shunt surgery is the “tap test” [5,17]. Nevertheless, a significant number of iNPH patients fail to respond. A reason for this may be the fact that iNPH often coexists with other neurodegenerative and/or vascular disorders, such as Alzheimer’s disease and vascular dementia (VD), known to present with similar clinical features. The latter also seems to share some pathophysiological mechanisms with iNPH. Krauss et al. (1996) found a significant association between iNPH and arterial hypertension, hypothesizing that arterial hypertension could be involved in the pathogenesis of iNPH [18]. Atherosclerosis could also play a role as it can increase systolic and pulse pressure via causing arterial stiffness, a fact that can eventually lead to ventricular enlargement [19,20].

Alzheimer’s disease (AD) is the most common neurodegenerative dementia entering in iNPH differential diagnosis [5,13,21,22]. Although AD and iNPH have long been considered distinct entities, recent studies suggest that they might share a common pathophysiologic mechanism [23,24]. Pathological studies have shown that the two disorders co-exist in a percentage up to 75% [25,26,27,28,29]. In the era of successful CSF biomarker development for AD, major interest is focused on how these biomarkers could be useful in studying co-occurrence of a neurodegenerative process, which could contribute to a better selection of patients as candidates for surgical treatment. There are a number of studies analyzing CSF biomarkers (established and emerging) in iNPH, although with contradictory results.

Therefore, we conducted the present narrative review, based on research and conceptual studies, in order to outline the general principles about the potential use of specific molecules as CSF biomarkers in patients with iNPH and their likely role in diagnosis, differential diagnosis and perhaps prognosis of this potentially reversible neurological disorder.

## 2. Materials and Methods

A search was performed to select studies in the PUBMED database from study inception to June 2022. There were no restrictions performed during the literature search. Keywords used to query the database included: “idiopathic normal pressure hydrocephalus”, “cerebrospinal fluid” and “biomarkers”, in all combinations. Editorials, case reports, case series, reviews, and case-control and cohort studies were also evaluated, and relevant information was selected. Duplicate publications were excluded from further assessment. Reference lists of all articles that met the criteria and references of relevant review articles were examined to identify studies that may have been missed by the database search. We summarized and synthesized the findings from the relevant articles and extracted their key findings.

## 3. Results

### 3.1. Biomarkers for Diagnosis and Differential Diagnosis of iNPH

#### 3.1.1. Established AD Biomarkers: Tau Proteins and Beta-Amyloid

Currently, the “AD CSF biomarker profile” is widely recognized and is characterized by increased concentrations of CSF tau proteins (total Tau protein and phosphorylated Tau protein), along with decreased concentration of CSF amyloid beta peptide with 42 amino acids (Aβ42) [30]. Total tau protein (t-tau) is generally considered a non-specific marker of neuronal/axonal degeneration, while phospho-tau (p-tau), mainly phosphorylated at a threonine residue at position 181, reflects tau hyperphosphorylation as well as tangle formation [31,32]. Aβ42 is indicative of the presence of amyloid pathology and correlates inversely with plaque burden [33].

Since the CSF amyloid and tau concentrations had already been established as diagnostic biomarkers in AD, the idea to explore their profile in NPH was reasonable. Kudo et al. (2000) were the first to report that CSF t-tau concentration in NPH patients was significantly higher than in controls [34]. Lins et al. (2004) found that CSF Aβ42 concentration was decreased in both NPH and AD patients, but similar (non-significantly different) between controls and patients with VD. They also reported that CSF t-tau concentration was increased in AD, but similar to controls in NPH and VD patients [35]. To our knowledge, the first study, analyzing all three CSF biomarkers in iNPH, was conducted by Kapaki et al. (2007), which showed that t-tau was significantly increased in iNPH and highly increased in AD as compared with the control group, whereas Aβ42 was decreased in both neurological diseases. CSF p-tau was significantly increased only in AD vs. healthy controls, but not in iNPH [36]. Agren-Wilsson et al. at the same time reported that CSF concentrations of t-tau, p-tau and Aβ42 were lower in iNPH compared to VD and healthy subjects [37], while Ray et al. (2011) showed a significant decrease in CSF Aβ42 concentration in NPH patients vs. healthy controls, but no significant difference in t-tau or p-tau between these two groups [38].

More recently, another study on iNPH was conducted by Jeppsson et al. (2013), which showed that patients with iNPH had low CSF concentrations of t-tau and p-tau and also low CSF concentrations of all the amyloid precursor protein (APP) fragments, including Aβ42. In addition, the authors of this study suggested that the concentrations of these proteins were lower in ventricular compared to lumbar CSF [39]. Miyajima et al. (2013) concluded that t-tau, p-tau and soluble amyloid precursor protein (sAPP) and its fragments (sAPPa and sAPPb) in CSF were lower in patients with iNPH than in patients with AD, while p-tau, sAPP, sAPPa and sAPPb CSF concentrations were lower in patients with iNPH than in healthy controls [40]. Tsai et al. (2014) found that p-tau was significantly lower in patients with iNPH than those with AD, using a univariate analysis, a difference that disappeared when multi-variate analysis was applied. Aβ42 and t-tau did not differ between groups [41].

Schirinzi et al. (2015) found that Aβ42 concentration was lower in iNPH than in healthy individuals but higher than in AD, and that t-tau and p-tau were lower in iNPH compared to AD [42]. Santangelo et al. (2017) reported that CSF Aβ42 concentration was lower in NPH patients than in controls but did not differ significantly compared to AD patients. However, according to the same study, t-tau and p-tau CSF concentrations were lower in NPH than in AD patients but did not differ significantly compared to controls [43]. Additionally, Jeppsson et al. (2019) showed that patients with iNPH had lower concentrations of t-tau and APP-derived proteins compared to patients with other causes of movement and dementing disorders and healthy individuals [44]. Taghdiri et al. (2020) suggested that lower concentrations of CSF Aβ42 and p-tau were observed in iNPH patients compared to controls. Lower concentrations of CSF p-tau and t-tau were also found in patients with iNPH compared to patients with AD, while CSF Aβ42 concentration was low in both groups. This study also highlighted a correlation of CSF-Aβ42 with age and Evans-index only in iNPH group [45].

Another proposed biomarker for amyloid pathology is Aβ42 toxic conformer ratio (Aβ42 toxic conformer/Aβ42 × 100). Aβ42 toxic conformer has a “toxic turn” structure at positions 22–23, causing quick β-sheet formation and thus neurotoxicity and synaptotoxicity. The aforementioned ratio has been reported higher among iNPH patients compared to healthy individuals and AD patients [46].

In an attempt to use CSF biomarkers to differentiate iNPH from subcortical ischemic vascular disease (SSVD), Manniche et al. (2020) suggested that lower concentrations of Aβ42 and t-tau were found in patients with iNPH compared to patients with subcortical vascular dementia, while concentration of p-tau in the CSF was similar in both diseases [47]. Said et al. (2022) suggested that in iNPH patients CSF concentration of Aβ42 was higher and those of t-tau and p-tau were lower than in AD patients [48], while Mazzeo et al. (2022) found that Aβ42/Aβ40, p-tau, and t-tau were significantly lower in iNPH than in AD and that Aβ42 concentration was similar in these two diseases [49]. Finally, all amyloid fragments’ CSF concentrations were found to be decreased in iNPH and SSVD compared to healthy individuals, with iNPH patients having lower concentrations than those with SSVD [50].

The differences of CSF concentrations of t-tau, p-tau and Aβ42, found in the above-mentioned studies, among iNPH patients and their discrepancies among healthy controls, patients with AD and patients with VD are summarized in Table 1.

#### 3.1.2. Emerging CSF Biomarkers in iNPH

Several other molecules related to neurodegeneration, neuroinflammation, demyelination and vascular pathology have been investigated as surrogate biomarkers.

Neurofilaments (NFs) are structural proteins consisting of three components with different molecular weight (high, medium or low) and degrees of phosphorylation [52]. Over the past two decades, an increasing number of studies have shown that neurofilament light chain (NFL) is associated with axonal injury or degeneration, and its CSF concentration has been found to be increased in various neurological disorders [53]. The majority of such studies have shown that iNPH patients exhibited higher CSF NFL concentration than healthy subjects [37,39,54,55,56,57], with the exception of Jeppsson et al. (2016), reporting that NFL concentration did not differ between iNPH patients and healthy individuals [58]. Lower concentrations of NFL in CSF have been reported in patients with iNPH compared with SSVD patients [47]; however, a more recent study concluded that NFL concentrations were similarly elevated in both diseases [50].

Another protein, proposed as a possible biomarker in NPH, is glial fibrillary acidic protein (GFAP). GFAP is the principal intermediate filament in mature astrocytes that seems to have a structural role in normal white matter architecture and blood–brain barrier integrity [59]. An early study reported increased CSF GFAP concentrations in NPH patients compared to patients with neurodegenerative dementia and healthy individuals [60]. However, two more recent studies, which compared GFAP concentrations in NPH patients and patients with subcortical arteriosclerotic encephalopathy, showed that there was no significant difference [50,61].

Furthermore, molecules related to neuroinflammation have been found increased in iNPH patients compared to healthy controls, such as interleukins (IL)-1β, IL-6 and IL-10, transforming growth factor (TGF)-β1 and TGFβ-type II receptor (TR-II). This later pathway might be involved in the pathogenesis of the disease, especially because of the direct correlation among TGF-β1, TR-II and LRG [62,63]. Leucine-rich-alpha-2-glykoprotein (LRG), a substance produced by astrocytes known to increase with aging and inflammation [64], have been found elevated in CSF of iNPH patients compared to healthy subjects [62,64,65,66]. Monocyte Chemoattractant Protein-1 (MCP-1), known to be over-expressed in glial cells during neuroinflammation or brain injury, was found increased in the CSF of iNPH patients compared to controls and patients with other neurological diseases [44].

Additionally, lipocalin-type prostaglandin D synthase (PGDS), also known as b-trace protein, considered to be mainly produced in the leptomeninges playing a role in prostaglandin metabolism and in retinoids’ transfer, has been found to have significantly lower concentration in CSF of NPH patients than in patients with other causes of dementia, such as AD, patients with depression and healthy individuals [67,68].

Myelin basic protein (MBP) is a structural component of the myelin sheath [69]. As an indicator of subcortical damage, it has been found to be increased in the CSF of iNPH patients [39,50,54]. Homocysteine is a thiol-containing amino acid, precursor of methionine whose metabolism depends mostly on co-factors, such as folate or vitamin B12 [70]. CSF concentration of homocysteine in iNPH patients was found significantly elevated compared to healthy subjects [71].

Proteins of the extracellular matrix (ECM) have also been studied in iNPH. ECM is produced by neurons and glial cells and is the material that “fills” the extracellular space. Typical components of the brain ECM are hyaluronic acid, heparan sulfate and chondroitin sulfate proteoglycans (including aggrecan, brevican, neurocan and versican), link proteins and glycoproteins [72]. The increase of ECM brevican and neurocan in CSF after shunt placement in iNPH might reflect normalization of the impaired ECM dynamics through restoration of CSF dynamics [73]. Matrix metalloproteinases (MMPs) are important for the morphogenesis and tissue repair caused by injury or by diseases [74]. MMP-10 has been found increased both in iNPH and SSVD compared to healthy individuals [50]. The fact that CSF MMPs-2 and -12 concentrations are found to be similar in patients with iNPH and patients with traumatic brain injury could mean that they reflect common pathways between iNPH and traumatic brain injury [75].

Transthyretin is a protein that acts mainly as a vehicle for the transport of thyroxin and retinol binding protein complex to different areas of the human body [76]. Its CSF concentration was found to be decreased only in AD and NPH and not in other causes of dementia by Gloeckner et al. (2008) [77]. Futakawa et al. (2012) showed that the ratio of two CSF transferrins (Tf), Tf-2/Tf-1 (Tf-1 with a unique N-glycan and Tf-2 with a N-glycan similar to that of serum transferrin), was significantly higher in iNPH compared to AD patients [78]. Furthermore, Nagata et al. in 2018 found significantly increased concentrations of serine and 2-hydroxybutyrate and significantly decreased concentrations of glycerate and N-acetylneuraminate in the CSF of iNPH patients compared to the CSF of AD patients [79].

Finally, CSF concentrations of protein tyrosine phosphatase receptor type Q (PTPRQ) in patients with iNPH were significantly higher than those of healthy individuals and those with Parkinson’s disease (PD) but did not differ significantly when compared to AD patients according to one study [80], while according to another study PTPRQ concentration in the CSF was significantly higher in patients with iNPH compared with those with AD [81].

### 3.2. Possible Correlations of Biomarker Concentrations with Severity of Symptoms, Prognosis, Tap Test and Shunt Responsiveness

The “gold standard” therapy for NPH is CSF diversion via a ventriculo-atrial or ventriculo-peritoneal shunt (VPS). Regarding established biomarkers, Agren-Wilsson et al. (2007) showed that t-tau and p-tau were significantly elevated after shunt surgery, whereas Aβ42 was not [37]. According to Jeppsson et al. (2013), all proteins were increased in the ventricular CSF except for t-tau, which was decreased [39]. Tullberg et al. (2008) concluded that there were no differences in any of the CSF biomarkers between patients that improved after surgery and those that did not [55]. In 2014, Pyykko et al. showed that sAPP was lower in shunt responsive iNPH than non-NPH patients [54].

However, there are some more recent studies suggesting that NPH patients who improved after shunt surgery showed a greater increase of APP-derived proteins in ventricular CSF after shunting than those who did not improve [39,82]. Golomb et al. (2000) concluded that concomitant AD pathology in iNPH patients does not significantly affect the clinical response to shunt surgery [27], while Lim et al. in 2014 found that concomitant AD pathology in iNPH patients might contribute to tap-test or shunt unresponsiveness, especially regarding cognitive impairment [83]. In 2020, Müller-Schmitz et al. concluded that iNPH patients with a CSF profile typical for AD improved significantly in cognition and gait following CSF removal, whereas iNPH patients without a typical AD profile did not, suggesting that patients with mixed iNPH–AD have a better prognosis than those with pure iNPH pathology [84]. Nevertheless, a most recent metanalysis made by Thavarajasingam et al. (2022), did not support the aforementioned conclusion, suggesting that CSF concentrations of t-tau and p-tau are significantly higher among iNPH patients that did not respond to shunt surgery compared to those who responded [85]. Finally, in 2022, Darrow et al. concluded that lower CSF concentrations of p-tau, t-tau and Aβ40 are associated with long-term improvement of kinetic symptoms [86].

Alterations in concentrations of various other substances, such as increased concentrations of a2HS glycoprotein, a1antichimotrypsin and a1beta glycoprotein, as well as decreased concentrations of GFAP, apolipoproteins (A-1, AIV, J and E), prostaglandin-H2 D-isomerase, Alpha-1-antitrypsin, serotransferrin complement C3c, anti-thrombin, a2 antiplasmin and albumin, have been reported to be associated with a positive response to surgery [87,88]. Furthermore, the PTPRQ concentration was lower in the CSF of non-responders to shunt operation compared with that in the CSF of responders [71].

Regarding the correlation of CSF biomarker concentrations to the severity of symptoms and the tap test responsiveness of iNPH patients, studies are scarce. Kang et al. (2014) found that lower CSF Aβ42 concentration correlated with worse cognitive impairment and lower CSF p-tau concentration correlated with gait difficulties. The CSF p-tau/Aβ-42 ratio was higher among CSF tap test non-responders compared to responders [89]. This is in contrast to the conclusion of Santangelo et al. (2017) that NPH patients with pathological Aβ42 concentration have the same cognitive performance with those with normal Aβ42 concentration [43]. As already mentioned, concentration of t-tau is connected to the severity of symptoms in NPH, suggesting that CSF t-tau may serve as a stage biological marker for NPH, useful to determine the level of neuronal degeneration [34]. Additionally, Ray et al. (2011) suggested that CSF p-tau concentration was significantly increased in the NPH patients with disease duration of more than one year and that the ratio of p-tau to Aβ42 may be a useful tool for predicting the possibility of NPH patients to also develop other neurodegenerative disorders including AD [38]. CSF p-tau has also been found to reflect cognitive prognosis, with increased concentrations correlating with cognitive deterioration two years postoperatively [46].

As regards NFL, higher concentrations were correlated with more extensive periventricular pathological density, worse gait, balance, wakefulness and neuropsychological performance, while lower concentrations were correlated to long-term kinetic improvement. Postoperatively, a greater improvement in gait and balance performance correlated with a greater reduction in NFL [55,56,86]. Despite the above, Jeppsson et al. (2013) concluded that NFL concentrations were increased after shunt surgery [39]. LRG is also considered a consistent indicator of brain damage in iNPH [64]. Finally, according to another study, the degree of improvement of iNPH symptoms correlated positively to the extent of the decrease of CSF homocysteine after CSF removal [71].

Summarized results of the aforementioned studies are shown in Table 2.

## 4. Discussion

While recognized for many years, CSF biomarkers have not been widely used in the diagnosis and management processes of iNPH. In this narrative review, we studied the available data from the English-speaking literature on the use of both established and emerging CSF biomarkers in diagnosis, differential diagnosis and possibly prognosis of iNPH patients.

Neurochemical diagnosis of iNPH relies so far on the established AD biomarkers, (t-tau and p-tau proteins and Aβ42 peptide), compared to healthy individuals. Research results from several studies agree on lower concentrations of Aβ42 and p-tau vs. controls [37,39,42,44,45,47], whereas t-tau concentration is either increased or decreased (Table 1). When comparing iNPH patients to AD patients, the majority of the studies agree with lower concentrations of t-tau and p-tau, whereas Aβ42 is either higher or similar/non-significantly different [36,37,38,39,41,42,45,48,54]. This is in accordance with the conclusions drown by Manniche et al. in 2019 that Aβ42 is lower in iNPH patients compared to healthy subjects but does not differ between iNPH and AD patients, whereas t-tau and p-tau are lower in iNPH than in AD [90]. Moreover, when comparing iNPH patients to patients with VD, the results, although rare, are suggestive of lower concentrations of t-tau and Aβ42 and inconclusive regarding the concentration of p-tau [37,44,47].

Aβ42 is well known to be implicated in the amyloid cascade hypothesis of AD, which states that the imbalance between the production and clearance of Aβ in the brain leads to neurodegeneration and cognitive impairment [91]. Patients with iNPH have decreased CSF concentrations of Aβ and sAPP fragments, sAPPα, sAPPβ. This is considered to be caused by a downregulation of APP in the periventricular parenchyma, possibly due to distorted amyloid metabolism and/or a decreased clearance of extracellular fluid into CSF [39]. Nevertheless, Aβ42, although considered a biomarker with molecular specificity for AD, has been also reported to be decreased in other neurodegenerative and non-degenerative disorders, such as Dementia with Lewy bodies, Frontotemporal dementia, VD and Creutzfeldt-Jakob disease [92,93,94,95,96], rendering Aβ42 a biomarker of low diagnostic power when used alone.

Phospho-tau on the other hand, is now considered a biomarker with molecular specificity for AD, reflecting neurofibrillary degeneration, and it is usually normal in other neurological diseases [92,93,97], including pure iNPH [37]. Increased CSF p-tau concentration reflects probably an AD coexistence as in mixed AD/VD dementia [36,98]. Total tau, on the contrary, is a non-specific marker of neurodegeneration/axonal damage like many other molecules, e.g., NFs, 14-3-3 protein and neuron specific enolase [99].

Although there are a good number of studies investigating AD biomarkers in iNPH, the data about other substances in the CSF of these patients with a potential diagnostic or prognostic role are relatively scarce. Differences in concentration of other CSF markers of neuronal damage, such as NFL, LRG, MBP and MCP-1 as well as many other CSF molecules have also been investigated in a limited number of studies, with contradictory results so far [37,39,54,55,56,57,62,64,65,66]. Thus, it is difficult to draw safe conclusions as per their suitability as diagnostic biomarkers for iNPH.

As regards the correlation of CSF biomarkers with the severity of symptoms, prognosis, tap test and shunt responsiveness, only a few indications can be highlighted. Greater increase of Aβ42 after shunt surgery seems to correlate positively with shunt responsiveness [39,82], even though Kang et al. in 2014 suggested that lower concentrations of Aβ42 were associated with worse cognitive performance [89]. Lower concentrations of t-tau, reflecting less neuronal damage, are correlated with less severe symptoms and greater possibility of kinetic improvement [34,86]. NFL concentration seems to positively correlate both with the severity of iNPH symptoms and the periventricular load of pathological densities, whereas the extent of reduction of its concentration after shunt surgery correlates positively with patients’ functional improvement [55,56].

We have to recognize the fact that there is a great heterogeneity among various such studies, regarding, in many cases, the inclusion criteria used for the characterization of the different patients’ cohorts, the methodology applied, including laboratory techniques, and the lack of appropriate control group for comparisons in many of such studies, thus making it difficult to reach firm conclusions.

In the era of personalized and precision medicine, coordinated efforts for the establishment of a unifying profile of CSF biomarkers would be very important, as it could not only shed more light on pathophysiological aspects of this complex neurologic syndrome and on concomitants pathologies, but could also become the basis for the timely and accurate diagnosis of this syndrome that would consequently lead to individualized treatment plans and better treatment outcomes

## 5. Conclusions

Most of the analyzed studies agree that iNPH patients present with lower concentrations of Aβ42 and p-tau than healthy individuals and lower concentrations of t-tau and p-tau than AD patients. New molecules are arising as possibly promising biomarkers, but more data regarding their sensitivity and specificity are definitely required. Thus, further studies are needed with strict inclusion criteria and well-standardized methodology to improve the value of CSF biomarkers in diagnosis, differential diagnosis and prognosis in iNPH, and to possibly contribute to a better selection of patients to submit for VPS.

## Figures and Tables

**Table 1:** CSF biomarker differences in iNPH versus healthy individuals, AD and VD patients.

iNPH			AD			VD			Reference
t-tau	p-tau	Aβ42	t-tau	p-tau	Aβ42	t-tau	p-tau	Aβ42	
↑	na	na	na	na	na	na	na	na	[34]
-	na	↓	↑	na	↓	-	na	-	[35]
↑	-	↓	↑↑	↑	↓	na	na	na	[36]
↓↓	↓↓	↓↓	na	na	na	↓	↓	↓	[37]
-	-/↑ *	↓	na	na	na	na	na	na	[38]
-	↓	-	↑	↑	-	na	na	na	[40]
↓	↓	↓	na	na	na	na	na	na	[39]
↑†	↓	↓	↑	↑	↓	na	na	na	[41]
↓†	↓	na	↑	↑	na	na	na	na	[51]
↓	↓	↓	↑	↑	↓↓	na	na	na	[42]
-	-	↓	↑	↑	↓	-	-	-	[43]
↓	↓	↓	↑↑	↑↑	↓↓	↑	↑	↓	[44]
↑	↓	↓	↑	↑	↓	na	na	na	[45]
↓↓	↓	↓↓	↑	↑	↓↓	↓	↓	↓	[47]
↓†	↓	↑	↑	↑	↓	na	na	na	[48]
na	na	↓↓	na	na	na	na	na	↓	[50]
↓†	↓	-	↑	↑	-	na	na	na	[49]

iNPH: idiopathic normal pressure hydrocephalus; AD: Alzheimer’s disease; VD: vascular dementia; t-tau: total tau protein; p-tau: phosphorylated tau protein; Aβ42: amyloid peptide with 42 amino acids; * in iNPH patients that have symptoms >1 year; na: non-applicable; - represents similar values; ↓ represents low values; ↓↓ represents lower values; ↑ represents high values; **↑↑** represents higher values; †: iNPH biomarkers are compared to healthy individuals except for these three lines in which iNPH profile is compared only to AD.

**Table 2:** CSF biomarkers correlations to severity of symptoms and prognosis.

CSF Biomarker	Clinical Correlation	Reference
t-tau high concentration	More severe symptoms	[34]
p-tau high concentration	Worse cognitive prognosis	[46,49]
p-tau high concentration	Longer disease duration (more than one year)	[38]
p-tau low concentration	Worse gait difficulties	[89]
APP-derived proteins increase in ventricular CSF	Shunt responder status	[39,82]
sAPP concentration	Lower in shunt responsive iNPH than non-NPH patients	[54]
Aβ42 concentrations low concentration	Worse cognitive impairment	[89]
Aβ42 concentration	No effect on cognitive prognosis	[43]
p-tau/Aβ42 high ratio	Shunt non-responder status	[89]
p-tau/Aβ42 high ratio	Higher possibility to develop a neurodegenerative disease	[38]
CSF biomarkers	No differences in any of them between responders and non-responders	[55]
AD profile	No effect on shunt responsiveness	[27]
AD profile	Negative effect to tap-test and shunt responsiveness	[83]
AD profile	Better gait and cognitive improvement after CSF removal	[84]
NFL high concentration	More extensive periventricular pathological density, worse: gait, balance, wakefulness and neuropsychological performance	[55,56,86]
NFL low concentration	Long term kinetic improvement	[55,56,86]
NFL reduction after shunt	Greater improvement in gait and balance	[55,56,86]
LRG high concentration	Brain damage	[64]
Homocysteine decrease after CSF removal	Greater symptoms improvement	[71]
a2HS glycoprotein, a1antichimotrypsin and a1beta glycoprotein increased concentration	Shunt responder status	[87,88]
GFAP, apolipoproteins (A-1, AIV, J and E), prostaglandin-H2 D-isomerase, Alpha-1-antitrypsin, serotransferrin complement C3c, anti-thrombin, a2 antiplasmin and albumin decreased concentration	Shunt responder status	[87,88]
PTQRP low concentration	Shunt non-responder status	[81]

CSF: cerebrospinal fluid; t-tau: total tau protein; p-tau: phosphorylated tau protein; APP: amyloid precursor protein; Aβ42: amyloid beta with 42 amino acids; AD: Alzheimer’s disease; NFL: neurofilament light chain; LRG: leucine-rich-alpha-2-glykoprotein; GFAP: glial fibrillary acidic protein; PTQRP: protein tyrosine phosphatase receptor type Q.

## Data Availability

Not applicable.

## References

[1] Rekate H.L. (2009). A contemporary definition and classification of hydrocephalus. Semin. Pediatr. Neurol..

[2] Leinonen V., Vanninen R., Rauramaa T. (2017). Cerebrospinal fluid circulation and hydrocephalus. Handb. Clin. Neurol..

[3] Ransohoff J., Shulman K., Fishman R.A. (1960). Hydrocephalus: A review of etiology and treatment. J. Pediatr..

[4] Oliveira L.M., Nitrini R., Roman G.C. (2019). Normal-pressure hydrocephalus: A critical review. Dement. Neuropsychol..

[5] Skalicky P., Mladek A., Vlasak A., De Lacy P., Benes V., Bradac O. (2020). Normal pressure hydrocephalus-an overview of pathophysiological mechanisms and diagnostic procedures. Neurosurg. Rev..

[6] Adams R.D., Fisher C.M., Hakim S., Ojemann R.G., Sweet W.H. (1965). Symptomatic occult hydrocephalus with normal cerebrospinal-fluid pressure: A treatable syndrome. N. Engl. J. Med..

[7] Hakim S., Adams R.D. (1965). The special clinical problem of symptomatic hydrocephalus with normal cerebrospinal fluid pressure. Observations on cerebrospinal fluid hydrodynamics. J. Neurol. Sci..

[8] Damasceno B.P. (2015). Neuroimaging in normal pressure hydrocephalus. Dement. Neuropsychol..

[9] Kockum K., Lilja-Lund O., Larsson E.M., Rosell M., Soderstrom L., Virhammar J., Laurell K. (2018). The idiopathic normal-pressure hydrocephalus Radscale: A radiological scale for structured evaluation. Eur. J. Neurol..

[10] Pyrgelis E.S., Paraskevas G.P., Constantinides V.C., Boufidou F., Velonakis G., Stefanis L., Kapaki E. (2022). Callosal Angle Sub-Score of the Radscale in Patients with Idiopathic Normal Pressure Hydrocephalus Is Associated with Positive Tap Test Response. J. Clin. Med..

[11] Relkin N., Marmarou A., Klinge P., Bergsneider M., Black P.M. (2005). Diagnosing idiopathic normal-pressure hydrocephalus. Neurosurgery.

[12] Kockum K., Larsson E.-M., Lilja-Lund O., Rosell M., Söderström L., Virhammar J., Laurell K. (2015). The NPH radscale; a new radiological scale for evaluation of suspected normal pressure hydrocephalus. Fluids Barriers CNS.

[13] Capone P.M., Bertelson J.A., Ajtai B. (2020). Neuroimaging of Normal Pressure Hydrocephalus and Hydrocephalus. Neurol. Clin..

[14] Mori E., Ishikawa M., Kato T., Kazui H., Miyake H., Miyajima M., Nakajima M., Hashimoto M., Kuriyama N., Tokuda T. (2012). Guidelines for management of idiopathic normal pressure hydrocephalus: Second edition. Neurol. Med. Chir..

[15] Bradley W.G. (2000). Normal pressure hydrocephalus: New concepts on etiology and diagnosis. AJNR. Am. J. Neuroradiol..

[16] Ammar A., Abbas F., Al Issawi W., Fakhro F., Batarfi L., Hendam A., Hasen M., El Shawarby M., Al Jehani H., Ammar A. (2017). Idiopathic Normal-Pressure Hydrocephalus Syndrome: Is It Understood? The Comprehensive Idiopathic Normal-Pressure Hydrocephalus Theory (CiNPHT). Hydrocephalus: What Do We Know? And What Do We Still Not Know?.

[17] Mihalj M., Dolic K., Kolic K., Ledenko V. (2016). CSF tap test—Obsolete or appropriate test for predicting shunt responsiveness? A systemic review. J. Neurol. Sci..

[18] Krauss J.K., Regel J.P., Vach W., Droste D.W., Borremans J.J., Mergner T. (1996). Vascular risk factors and arteriosclerotic disease in idiopathic normal-pressure hydrocephalus of the elderly. Stroke.

[19] Graff-Radford N.R., Knopman D.S., Penman A.D., Coker L.H., Mosley T.H. (2013). Do systolic BP and pulse pressure relate to ventricular enlargement?. Eur. J. Neurol..

[20] Hooglugt A., Klatt O., Huveneers S. (2022). Vascular stiffening and endothelial dysfunction in atherosclerosis. Curr. Opin. Lipidol..

[21] Kitagaki H., Mori E., Ishii K., Yamaji S., Hirono N., Imamura T. (1998). CSF spaces in idiopathic normal pressure hydrocephalus: Morphology and volumetry. AJNR. Am. J. Neuroradiol..

[22] Siraj S. (2011). An overview of normal pressure hydrocephalus and its importance: How much do we really know?. J. Am. Med. Dir. Assoc..

[23] Silverberg G.D., Mayo M., Saul T., Rubenstein E., McGuire D. (2003). Alzheimer’s disease, normal-pressure hydrocephalus, and senescent changes in CSF circulatory physiology: A hypothesis. Lancet. Neurol..

[24] Silverberg G.D., Mayo M., Saul T., Fellmann J., Carvalho J., McGuire D. (2008). Continuous CSF drainage in AD: Results of a double-blind, randomized, placebo-controlled study. Neurology.

[25] Del Bigio M.R., Cardoso E.R., Halliday W.C. (1997). Neuropathological changes in chronic adult hydrocephalus: Cortical biopsies and autopsy findings. Can. J. Neurol. Sci..

[26] Savolainen S., Paljarvi L., Vapalahti M. (1999). Prevalence of Alzheimer’s disease in patients investigated for presumed normal pressure hydrocephalus: A clinical and neuropathological study. Acta Neurochir..

[27] Golomb J., Wisoff J., Miller D.C., Boksay I., Kluger A., Weiner H., Salton J., Graves W. (2000). Alzheimer’s disease comorbidity in normal pressure hydrocephalus: Prevalence and shunt response. J. Neurol. Neurosurg. Psychiatry.

[28] Bech-Azeddine R., Hogh P., Juhler M., Gjerris F., Waldemar G. (2007). Idiopathic normal-pressure hydrocephalus: Clinical comorbidity correlated with cerebral biopsy findings and outcome of cerebrospinal fluid shunting. J. Neurol. Neurosurg. Psychiatry.

[29] Leinonen V., Alafuzoff I., Aalto S., Suotunen T., Savolainen S., Nagren K., Tapiola T., Pirttila T., Rinne J., Jaaskelainen J.E. (2008). Assessment of beta-amyloid in a frontal cortical brain biopsy specimen and by positron emission tomography with carbon 11-labeled Pittsburgh Compound B. Arch. Neurol..

[30] Biscetti L., Salvadori N., Farotti L., Cataldi S., Eusebi P., Paciotti S., Parnetti L. (2019). The added value of Abeta42/Abeta40 in the CSF signature for routine diagnostics of Alzheimer’s disease. Clin. Chim. Acta Int. J. Clin. Chem..

[31] Blennow K., Hampel H. (2003). CSF markers for incipient Alzheimer’s disease. Lancet. Neurol..

[32] van Harten A.C., Kester M.I., Visser P.J., Blankenstein M.A., Pijnenburg Y.A., van der Flier W.M., Scheltens P. (2011). Tau and p-tau as CSF biomarkers in dementia: A meta-analysis. Clin. Chem. Lab. Med..

[33] Murphy M.P., LeVine H. (2010). Alzheimer’s disease and the amyloid-beta peptide. J. Alzheimer’s Dis. JAD.

[34] Kudo T., Mima T., Hashimoto R., Nakao K., Morihara T., Tanimukai H., Tsujio I., Koike Y., Tagami S., Mori H. (2000). Tau protein is a potential biological marker for normal pressure hydrocephalus. Psychiatry Clin. Neurosci..

[35] Lins H., Wichart I., Bancher C., Wallesch C.W., Jellinger K.A., Rosler N. (2004). Immunoreactivities of amyloid beta peptide((1-42)) and total tau protein in lumbar cerebrospinal fluid of patients with normal pressure hydrocephalus. J. Neural Transm..

[36] Kapaki E.N., Paraskevas G.P., Tzerakis N.G., Sfagos C., Seretis A., Kararizou E., Vassilopoulos D. (2007). Cerebrospinal fluid tau, phospho-tau181 and beta-amyloid1-42 in idiopathic normal pressure hydrocephalus: A discrimination from Alzheimer’s disease. Eur. J. Neurol..

[37] Agren-Wilsson A., Lekman A., Sjoberg W., Rosengren L., Blennow K., Bergenheim A.T., Malm J. (2007). CSF biomarkers in the evaluation of idiopathic normal pressure hydrocephalus. Acta Neurol. Scand..

[38] Ray B., Reyes P.F., Lahiri D.K. (2011). Biochemical studies in Normal Pressure Hydrocephalus (NPH) patients: Change in CSF levels of amyloid precursor protein (APP), amyloid-beta (Abeta) peptide and phospho-tau. J. Psychiatr. Res..

[39] Jeppsson A., Zetterberg H., Blennow K., Wikkelso C. (2013). Idiopathic normal-pressure hydrocephalus: Pathophysiology and diagnosis by CSF biomarkers. Neurology.

[40] Miyajima M., Nakajima M., Ogino I., Miyata H., Motoi Y., Arai H. (2013). Soluble amyloid precursor protein alpha in the cerebrospinal fluid as a diagnostic and prognostic biomarker for idiopathic normal pressure hydrocephalus. Eur. J. Neurol..

[41] Tsai A., Malek-Ahmadi M., Kahlon V., Sabbagh M.N. (2014). Differences in Cerebrospinal Fluid Biomarkers between Clinically Diagnosed Idiopathic Normal Pressure Hydrocephalus and Alzheimer’s Disease. J. Alzheimer’s Dis. Park..

[42] Schirinzi T., Sancesario G.M., Ialongo C., Imbriani P., Madeo G., Toniolo S., Martorana A., Pisani A. (2015). A clinical and biochemical analysis in the differential diagnosis of idiopathic normal pressure hydrocephalus. Front. Neurol..

[43] Santangelo R., Cecchetti G., Bernasconi M.P., Cardamone R., Barbieri A., Pinto P., Passerini G., Scomazzoni F., Comi G., Magnani G. (2017). Cerebrospinal Fluid Amyloid-beta 42, Total Tau and Phosphorylated Tau are Low in Patients with Normal Pressure Hydrocephalus: Analogies and Differences with Alzheimer’s Disease. J. Alzheimer’s Dis. JAD.

[44] Jeppsson A., Wikkelso C., Blennow K., Zetterberg H., Constantinescu R., Remes A.M., Herukka S.K., Rauramaa T., Nagga K., Leinonen V. (2019). CSF biomarkers distinguish idiopathic normal pressure hydrocephalus from its mimics. J. Neurol. Neurosurg. Psychiatry.

[45] Taghdiri F., Gumus M., Algarni M., Fasano A., Tang-Wai D., Tartaglia M.C. (2020). Association Between Cerebrospinal Fluid Biomarkers and Age-related Brain Changes in Patients with Normal Pressure Hydrocephalus. Sci. Rep..

[46] Akiba C., Nakajima M., Miyajima M., Ogino I., Motoi Y., Kawamura K., Adachi S., Kondo A., Sugano H., Tokuda T. (2018). Change of Amyloid-beta 1-42 Toxic Conformer Ratio After Cerebrospinal Fluid Diversion Predicts Long-Term Cognitive Outcome in Patients with Idiopathic Normal Pressure Hydrocephalus. J. Alzheimer’s Dis. JAD.

[47] Manniche C., Simonsen A.H., Hasselbalch S.G., Andreasson U., Zetterberg H., Blennow K., Hogh P., Juhler M., Hejl A.M. (2020). Cerebrospinal Fluid Biomarkers to Differentiate Idiopathic Normal Pressure Hydrocephalus from Subcortical Ischemic Vascular Disease. J. Alzheimer’s Dis. JAD.

[48] Said H.M., Kaya D., Yavuz I., Dost F.S., Altun Z.S., Isik A.T. (2022). A Comparison of Cerebrospinal Fluid Beta-Amyloid and Tau in Idiopathic Normal Pressure Hydrocephalus and Neurodegenerative Dementias. Clin. Interv. Aging.

[49] Mazzeo S., Emiliani F., Bagnoli S., Padiglioni S., Del Re L.M., Giacomucci G., Balestrini J., Ingannato A., Moschini V., Morinelli C. (2022). Alzheimer’s Disease CSF Biomarker Profiles in Idiopathic Normal Pressure Hydrocephalus. J. Pers. Med..

[50] Jeppsson A., Bjerke M., Hellstrom P., Blennow K., Zetterberg H., Kettunen P., Wikkelso C., Wallin A., Tullberg M. (2022). Shared CSF Biomarker Profile in Idiopathic Normal Pressure Hydrocephalus and Subcortical Small Vessel Disease. Front. Neurol..

[51] Jingami N., Asada-Utsugi M., Uemura K., Noto R., Takahashi M., Ozaki A., Kihara T., Kageyama T., Takahashi R., Shimohama S. (2015). Idiopathic normal pressure hydrocephalus has a different cerebrospinal fluid biomarker profile from Alzheimer's disease. J. Alzheimer'sDis. JAD.

[52] Hoffman P.N., Cleveland D.W., Griffin J.W., Landes P.W., Cowan N.J., Price D.L. (1987). Neurofilament gene expression: A major determinant of axonal caliber. Proc. Natl. Acad. Sci. USA.

[53] Gaetani L., Blennow K., Calabresi P., Di Filippo M., Parnetti L., Zetterberg H. (2019). Neurofilament light chain as a biomarker in neurological disorders. J. Neurol. Neurosurg. Psychiatry.

[54] Pyykko O.T., Lumela M., Rummukainen J., Nerg O., Seppala T.T., Herukka S.K., Koivisto A.M., Alafuzoff I., Puli L., Savolainen S. (2014). Cerebrospinal fluid biomarker and brain biopsy findings in idiopathic normal pressure hydrocephalus. PLoS ONE.

[55] Tullberg M., Blennow K., Mansson J.E., Fredman P., Tisell M., Wikkelso C. (2008). Cerebrospinal fluid markers before and after shunting in patients with secondary and idiopathic normal pressure hydrocephalus. Cereb. Fluid Res..

[56] Tullberg M., Blennow K., Mansson J.E., Fredman P., Tisell M., Wikkelso C. (2007). Ventricular cerebrospinal fluid neurofilament protein levels decrease in parallel with white matter pathology after shunt surgery in normal pressure hydrocephalus. Eur. J. Neurol..

[57] Tullberg M., Rosengren L., Blomsterwall E., Karlsson J.E., Wikkelso C. (1998). CSF neurofilament and glial fibrillary acidic protein in normal pressure hydrocephalus. Neurology.

[58] Jeppsson A., Holtta M., Zetterberg H., Blennow K., Wikkelso C., Tullberg M. (2016). Amyloid mis-metabolism in idiopathic normal pressure hydrocephalus. Fluids Barriers CNS.

[59] Eng L.F., Ghirnikar R.S., Lee Y.L. (2000). Glial fibrillary acidic protein: GFAP-thirty-one years (1969–2000). Neurochem. Res..

[60] Albrechtsen M., Sorensen P.S., Gjerris F., Bock E. (1985). High cerebrospinal fluid concentration of glial fibrillary acidic protein (GFAP) in patients with normal pressure hydrocephalus. J. Neurol. Sci..

[61] Tullberg M., Mansson J.E., Fredman P., Lekman A., Blennow K., Ekman R., Rosengren L.E., Tisell M., Wikkelso C. (2000). CSF sulfatide distinguishes between normal pressure hydrocephalus and subcortical arteriosclerotic encephalopathy. J. Neurol. Neurosurg. Psychiatry.

[62] Li X., Miyajima M., Jiang C., Arai H. (2007). Expression of TGF-betas and TGF-beta type II receptor in cerebrospinal fluid of patients with idiopathic normal pressure hydrocephalus. Neurosci. Lett..

[63] Sosvorova L., Mohapl M., Vcelak J., Hill M., Vitku J., Hampl R. (2015). The impact of selected cytokines in the follow-up of normal pressure hydrocephalus. Physiol. Res..

[64] Nakajima M., Miyajima M., Ogino I., Watanabe M., Miyata H., Karagiozov K.L., Arai H., Hagiwara Y., Segawa T., Kobayashi K. (2011). Leucine-rich alpha-2-glycoprotein is a marker for idiopathic normal pressure hydrocephalus. Acta Neurochir..

[65] Nakajima M., Arai H., Miyajima M. (2010). Diagnostic value of CSF biomarker profile in idiopathic normal pressure hydrocephalus; leucine-rich alpha-2-glycoprotein is a potential biological marker. Rinsho Shinkeigaku = Clin. Neurol..

[66] Li X., Miyajima M., Mineki R., Taka H., Murayama K., Arai H. (2006). Analysis of potential diagnostic biomarkers in cerebrospinal fluid of idiopathic normal pressure hydrocephalus by proteomics. Acta Neurochir..

[67] Mase M., Yamada K., Shimazu N., Seiki K., Oda H., Nakau H., Inui T., Li W., Eguchi N., Urade Y. (2003). Lipocalin-type prostaglandin D synthase (beta-trace) in cerebrospinal fluid: A useful marker for the diagnosis of normal pressure hydrocephalus. Neurosci. Res..

[68] Brettschneider J., Riepe M.W., Petereit H.F., Ludolph A.C., Tumani H. (2004). Meningeal derived cerebrospinal fluid proteins in different forms of dementia: Is a meningopathy involved in normal pressure hydrocephalus?. J. Neurol. Neurosurg. Psychiatry.

[69] Omlin F.X., Webster H.D., Palkovits C.G., Cohen S.R. (1982). Immunocytochemical localization of basic protein in major dense line regions of central and peripheral myelin. J. Cell Biol..

[70] Selhub J., Miller J.W. (1992). The pathogenesis of homocysteinemia: Interruption of the coordinate regulation by S-adenosylmethionine of the remethylation and transsulfuration of homocysteine. Am. J. Clin. Nutr..

[71] Sosvorova L., Bestak J., Bicikova M., Mohapl M., Hill M., Kubatova J., Hampl R. (2014). Determination of homocysteine in cerebrospinal fluid as an indicator for surgery treatment in patients with hydrocefalus. Physiol. Res..

[72] Frischknecht R., Chang K.J., Rasband M.N., Seidenbecher C.I. (2014). Neural ECM molecules in axonal and synaptic homeostatic plasticity. Prog. Brain Res..

[73] Minta K., Jeppsson A., Brinkmalm G., Portelius E., Zetterberg H., Blennow K., Tullberg M., Andreasson U. (2021). Lumbar and ventricular CSF concentrations of extracellular matrix proteins before and after shunt surgery in idiopathic normal pressure hydrocephalus. Fluids Barriers CNS.

[74] Simoes G., Pereira T., Caseiro A. (2022). Matrix metaloproteinases in vascular pathology. Microvasc. Res..

[75] Minta K., Brinkmalm G., Al Nimer F., Thelin E.P., Piehl F., Tullberg M., Jeppsson A., Portelius E., Zetterberg H., Blennow K. (2020). Dynamics of cerebrospinal fluid concentrations of matrix metalloproteinases in human traumatic brain injury. Sci. Rep..

[76] Sharma M., Khan S., Rahman S., Singh L.R. (2019). The Extracellular Protein, Transthyretin Is an Oxidative Stress Biomarker. Front. Physiol..

[77] Gloeckner S.F., Meyne F., Wagner F., Heinemann U., Krasnianski A., Meissner B., Zerr I. (2008). Quantitative analysis of transthyretin, tau and amyloid-beta in patients with dementia. J. Alzheimer’s Dis. JAD.

[78] Futakawa S., Nara K., Miyajima M., Kuno A., Ito H., Kaji H., Shirotani K., Honda T., Tohyama Y., Hoshi K. (2012). A unique N-glycan on human transferrin in CSF: A possible biomarker for iNPH. Neurobiol. Aging.

[79] Nagata Y., Hirayama A., Ikeda S., Shirahata A., Shoji F., Maruyama M., Kayano M., Bundo M., Hattori K., Yoshida S. (2018). Comparative analysis of cerebrospinal fluid metabolites in Alzheimer’s disease and idiopathic normal pressure hydrocephalus in a Japanese cohort. Biomark. Res..

[80] Nakajima M., Rauramaa T., Makinen P.M., Hiltunen M., Herukka S.K., Kokki M., Musialowicz T., Jyrkkanen H.K., Danner N., Junkkari A. (2021). Protein tyrosine phosphatase receptor type Q in cerebrospinal fluid reflects ependymal cell dysfunction and is a potential biomarker for adult chronic hydrocephalus. Eur. J. Neurol..

[81] Nagata Y., Bundo M., Sugiura S., Kamita M., Ono M., Hattori K., Yoshida S., Goto Y.I., Urakami K., Niida S. (2017). PTPRQ as a potential biomarker for idiopathic normal pressure hydrocephalus. Mol. Med. Rep..

[82] Graff-Radford N.R., Jones D.T. (2019). Normal Pressure Hydrocephalus. Contin. Lifelong Learn. Neurol..

[83] Lim T.S., Choi J.Y., Park S.A., Youn Y.C., Lee H.Y., Kim B.G., Joo I.S., Huh K., Moon S.Y. (2014). Evaluation of coexistence of Alzheimer’s disease in idiopathic normal pressure hydrocephalus using ELISA analyses for CSF biomarkers. BMC Neurol..

[84] Muller-Schmitz K., Krasavina-Loka N., Yardimci T., Lipka T., Kolman A.G.J., Robbers S., Menge T., Kujovic M., Seitz R.J. (2020). Normal Pressure Hydrocephalus Associated with Alzheimer’s Disease. Ann. Neurol..

[85] Thavarajasingam S.G., El-Khatib M., Vemulapalli K.V., Iradukunda H.A.S., Laleye J., Russo S., Eichhorn C., Eide P.K. (2022). Cerebrospinal fluid and venous biomarkers of shunt-responsive idiopathic normal pressure hydrocephalus: A systematic review and meta-analysis. Acta Neurochir..

[86] Darrow J.A., Lewis A., Gulyani S., Khingelova K., Rao A., Wang J., Zhang Y., Luciano M., Yasar S., Moghekar A. (2022). CSF Biomarkers Predict Gait Outcomes in Idiopathic Normal Pressure Hydrocephalus. Neurol. Clin. Pract..

[87] Chen C.P.C., Huang Y.C., Chang C.N., Chen J.L., Hsu C.C., Lin W.Y. (2018). Changes of cerebrospinal fluid protein concentrations and gait patterns in geriatric normal pressure hydrocephalus patients after ventriculoperitoneal shunting surgery. Exp. Gerontol..

[88] Scollato A., Terreni A., Caldini A., Salvadori B., Gallina P., Francese S., Mastrobuoni G., Pieraccini G., Moneti G., Bini L. (2010). CSF proteomic analysis in patients with normal pressure hydrocephalus selected for the shunt: CSF biomarkers of response to surgical treatment. Neurol. Sci. Off. J. Ital. Neurol. Soc. Ital. Soc. Clin. Neurophysiol..

[89] Kang K., Ko P.W., Jin M., Suk K., Lee H.W. (2014). Idiopathic normal-pressure hydrocephalus, cerebrospinal fluid biomarkers, and the cerebrospinal fluid tap test. J. Clin. Neurosci. Off. J. Neurosurg. Soc. Australas..

[90] Manniche C., Hejl A.M., Hasselbalch S.G., Simonsen A.H. (2019). Cerebrospinal Fluid Biomarkers in Idiopathic Normal Pressure Hydrocephalus versus Alzheimer’s Disease and Subcortical Ischemic Vascular Disease: A Systematic Review. J. Alzheimer’s Dis. JAD.

[91] Blennow K., de Leon M.J., Zetterberg H. (2006). Alzheimer’s disease. Lancet.

[92] Kapaki E., Paraskevas G.P., Papageorgiou S.G., Bonakis A., Kalfakis N., Zalonis I., Vassilopoulos D. (2008). Diagnostic value of CSF biomarker profile in frontotemporal lobar degeneration. Alzheimer Dis. Assoc. Disord..

[93] Paraskevas G.P., Kapaki E., Papageorgiou S.G., Kalfakis N., Andreadou E., Zalonis I., Vassilopoulos D. (2009). CSF biomarker profile and diagnostic value in vascular dementia. Eur. J. Neurol..

[94] Lewczuk P., Riederer P., O’Bryant S.E., Verbeek M.M., Dubois B., Visser P.J., Jellinger K.A., Engelborghs S., Ramirez A., Parnetti L. (2018). Cerebrospinal fluid and blood biomarkers for neurodegenerative dementias: An update of the Consensus of the Task Force on Biological Markers in Psychiatry of the World Federation of Societies of Biological Psychiatry. World J. Biol. Psychiatry Off. J. World Fed. Soc. Biol. Psychiatry.

[95] Paraskevas G.P., Bougea A., Constantinides V.C., Bourbouli M., Petropoulou O., Kapaki E. (2019). In vivo Prevalence of Alzheimer Biomarkers in Dementia with Lewy Bodies. Dement. Geriatr. Cogn. Disord..

[96] Kokkinou M., Beishon L.C., Smailagic N., Noel-Storr A.H., Hyde C., Ukoumunne O., Worrall R.E., Hayen A., Desai M., Ashok A.H. (2021). Plasma and cerebrospinal fluid ABeta42 for the differential diagnosis of Alzheimer’s disease dementia in participants diagnosed with any dementia subtype in a specialist care setting. Cochrane Database Syst. Rev..

[97] Jack C.R., Bennett D.A., Blennow K., Carrillo M.C., Feldman H.H., Frisoni G.B., Hampel H., Jagust W.J., Johnson K.A., Knopman D.S. (2016). A/T/N: An unbiased descriptive classification scheme for Alzheimer disease biomarkers. Neurology.

[98] Wallin A., Kapaki E., Boban M., Engelborghs S., Hermann D.M., Huisa B., Jonsson M., Kramberger M.G., Lossi L., Malojcic B. (2017). Biochemical markers in vascular cognitive impairment associated with subcortical small vessel disease—A consensus report. BMC Neurol..

[99] Jack C.R., Bennett D.A., Blennow K., Carrillo M.C., Dunn B., Haeberlein S.B., Holtzman D.M., Jagust W., Jessen F., Karlawish J. (2018). NIA-AA Research Framework: Toward a biological definition of Alzheimer’s disease. Alzheimer’s Dement. J. Alzheimer’s Assoc..

